# Prevalence, Clinical Manifestations and Genotyping of *Cryptosporidium* Spp. in Patients with Gastrointestinal Illnesses in Western Iran

**Published:** 2017

**Authors:** Hamed KIANI, Ali HAGHIGHI, Seyyed Javad SEYYEDTABAEI, Eznollah AZARGASHSB, Nozhat ZEBARDAST, Niloofar TAGHIPOUR, Ali ROSTAMI, Lihua XIAO

**Affiliations:** 1. Dept. of Medical Parasitology and Mycology, School of Medicine, Shahid Beheshti University of Medical Sciences, Tehran, Iran; 2. Iranian Veterinary Organization, Hamadan, Iran; 3. Dept. of Community Medicine, School of Medicine, Shahid Beheshti University of Medical Sciences, Tehran, Iran; 4. Student Research Committee, Shahid Beheshti University of Medical Sciences, Tehran, Iran; 5. Division of Foodborne, Waterborne and Environmental Diseases, National Center for Emerging and Zoonotic Infectious Diseases, Centers for Disease Control and Prevention, Public Health Services, Atlanta, U.S.A

**Keywords:** Cryptosporidiosis, Clinical manifestations, Genotyping, Gastrointestinal illnesses, Iran

## Abstract

**Background:**

*Cryptosporidium* species are recognized as important gastrointestinal pathogens. This study was conducted to identify the prevalence, clinical manifestations and genotyping of *Cryptosporidium* spp. in patients with gastrointestinal illnesses (GIs) in western Iran.

**Methods:**

Overall, 1301 fecal samples were collected from patients with GIs referred to the 12 clinical laboratories in Nahavand County, west of Iran. Modified Ziehl-Neelsen staining method was used to identify the oocysts. DNA was extracted from positive samples and *Cryptosporidium* spp. were characterized by Nested PCR and sequence analysis of the 60-kDa glycoprotein (gp60) gene. Data analysis was performed using SPSS ver. 16.

**Results:**

Prevalence of cryptosporidiosis was 1.3% (17/1301). *Cryptosporidium* infection was significantly associated with vomiting and nausea (*P*=0.001, OR=0.013; CI 95%=0.004– 0.044), abdominal pain (*P*=0.018, OR=0.073; CI 95%=0.008– 0.633) and diarrhea (*P*=0.001, OR=0.092; CI 95%=0.023– 0.362). Of the 17 isolates typed, 11 belonged to the *C. parvum* IId subtype family (subtypes IIdA26G1 and IIdA20G1) and six belonged to the *C. parvum* IIa subtype family (subtypes IIaA15G2R1 and IIaA16G3R1). There was no significant difference between sub-type families IIa and IId in occurrence of clinical symptoms (*P*= 0.75).

**Conclusion:**

Improved hygiene and avoidance of contact with animals and contaminated soil should be advocated to reduce the occurrence of *Cryptosporidium* infections, especially in children.

## Introduction

A cute gastrointestinal illnesses (AGIs) are major causes of hospitalization throughout the world. In developing countries, AGIs are one of the leading causes of morbidity and mortality (1). The most common symptoms of gastrointestinal illnesses (GI) are diarrhea, abdominal pain, and vomiting. Diarrhea is the second leading cause of deaths among children less than five years of age, especially in low and middle-income countries (2). Intestinal protozoan and helminthic infections are among leading causes of gastrointestinal disorders (3, 4).

Protozoa of the genus *Cryptosporidium* are recognized as important gastrointestinal pathogens that infect a wide range of vertebrates including humans. *Cryptosporidium* spp. are well adapted to zoonotic, waterborne and food-borne transmission, and transmitted to hosts by the fecal-oral route (5). *Cryptosporidium* spp. can cause a wide spectrum of symptoms, from severe life-threatening diarrhea or vomiting in immunocompromised patients to asymptomatic and self-limiting infection in immunocompetent individuals (4, 6).

*C. hominis* and *C. parvum* are the most common etiologic agents of human cryptosporidiosis worldwide, and the latter is commonly responsible for zoonotic infections (7). Other reported zoonotic *Cryptosporidium* species include *C. meleagridis*, *C. felis*, *C. muris*, *C. canis,* and C. *ubiquitum* (8, 9).

A variety of molecular methods has been used for differentiation of *Cryptosporidium* species/genotypes and *C. parvum* and *C. hominis* subtypes. Subtyping tools have been used extensively in studies of the transmission of *C. hominis* in humans and *C. parvum* in humans and ruminants (9). The DNA sequence analysis of 60-kDa glycoprotein gene (gp60) is currently the most widely used genetic marker in studies of the host adaptation, genetic diversity, transmission dynamics and infection sources of *Cryptosporidium* spp. (8, 9). The gp60 subtyping showed that *C. parvum* had 12 sub-type families (IIa–IIl) and subtype families IIa and IId are considered major zoonotic ones, whilst IIc subtype family considered the major anthroponotic one. *C. hominis* has been polymorphic and has at least seven subtype families (Ia–Ig) (9, 10). Several molecular and epidemiological studies in Iran have demonstrated moderate prevalence of *Cryptosporidium* spp. in different populations and have shown that *C. parvum* is the predominant species in human and livestock (4, 11–13).

The main aim of the present study was to evaluate the occurrence, clinical manifestations and subtypes of *Cryptosporidium* spp. in patients with acute gastrointestinal illnesses in Nahavand County, western Iran.

## Materials and Methods

### Study area and population

This cross-sectional study was conducted from Apr to Sept 2014 in 1301 patients with GIs referred to the 12 clinical laboratories in Nahavand County, west of Iran. Patients not given any anti-parasitic drugs in the week prior to the study were included in this study. A questionnaire survey was administered to each participant focusing on demography (age, gender, and location), gastrointestinal symptoms (abdominal pain, cramping, bloating, vomiting & nausea, diarrhea, dysentery, and constipation), living condition and water usage.

### Microscopy of stool specimens

After completing the questionnaire, all participants were given a clean and dry plastic container pre-labeled with their identification numbers. The fecal specimens were examined microscopically to determine the consistency, presence of blood and mucus and any other abnormalities. To identify oocysts of *Cryptosporidium* spp., a permanent slide was prepared for each sample after oocyst concentration with the formaldehyde-diethyl ether centrifugation method, and stained with the modified Ziehl–Neelsen acid-fast technique, as described previously (14). Samples with excessive mucus were smeared directly and stained without concentration technique. The stained smears were examined under a microscope (Zeiss, Germany, 100× magnification). All positive *Cryptosporidium* specimens were stored in 70% ethanol for DNA extraction.

### DNA extraction

Extraction of genomic DNA was performed using 100 mg of stool specimens and the DNA isolation stool mini kit (Yekta Tajhiz Azma Co., Iran) according to the manufacturer’s instructions, after washing of specimens three times with phosphate buffered saline (PBS) by centrifugation at 14000 rpm for 4 min. The extracted DNA was stored at −20°C until PCR analysis.

### PCR Amplification

A ∼400-bp fragment of the gp60 gene was amplified by nested PCR using the primer sets 5′-ATAGTCTCCGCTGTATTC-3′ and 5′-GCA GAGGAACCAGCATC-3′ in the primary PCR and 5′-TCCGCTGTATTCTCAGCC-3′ and 5′-GAGATATATCTTGGTGCG-3′ in the secondary PCR, as described previously (13). The PCR was performed using the Taq DNA Polymerase Master Mix Red (Amplicon, Denmark). The reaction mixture contained 5 μl distilled water, 7.5 μl master mix, 20 pmol forward and reverse primers and about 25-100 ng/μl of extracted DNA in a final volume of 15 μl. DNA from a known *Cryptosporidium* species and a blank containing all PCR reagents but no DNA were included in each set of PCR as positive and negative controls, respectively. PCR products were visualized by electrophoresis on 1.5% agarose gels stained with ethidium bromide.

### DNA sequence analysis

Products of the secondary PCR were sequenced in using Applied Biosystems 3730/3730×l DNA Analyzers (Bioneer, Korea). All sequences were assembled and edited manually using the Chromas program version 1.0.0.1. Basic Local Alignment Search Tool (BLAST) was used to analyze sequences obtained from this study against data in Gen-Bank. The established subtype nomemclature was used in naming *C. parvum* subtypes (9).

### Statistical analysis

Data from the study were analyzed using the SPSS software version 16 (SPSS, Chicago, IL, USA). Categorical variables are presented as frequencies and percentage. Logistic regression analysis was used to identify potential risk factors for cryptosporidiosis occurrence. Associations were tested using odds ratios (OR) and 95% confidence intervals (CI) after adjustments. *P* values <0.05 were considered statistically significant.

### Ethical Considerations

All procedures in this study were approved by the Ethics Committee of the Shahid Beheshti University of Medical Science, before the beginning of the study (Grant. No. 13/1285). All study participants were informed about the study procedures and written informed consents were obtained from all of them prior to sample collection.

## Results

### Occurrence of cryptosporidiosis

Overall, 1301 GIs patients, 619 (47.6%) were female, 682 (52.4%) male. The median age of the study participants was 26 yr (range: 22 d to 90 yr). The prevalence of cryptosporidiosis among patients was 1.3% (17/1301).

### Cryptosporidium genotypes and subtypes

**Fig. 1:**
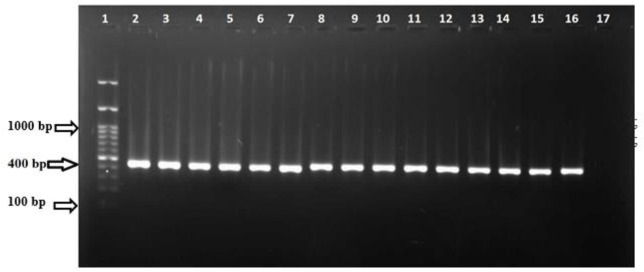
Identification of *Cryptosporidium* species using Nested PCR. Lan 1, DNA Marker (100 bp,); Lan 2–15, DNA samples amplified with 60-kDa glycoprotein (gp60) gene amplimer pairs (400 bp); Lan 16, positive control sample; Lan 17, negative control

Species identification by nested PCR was successful for all 17 *Cryptosporidium*-positive specimens (Fig. 1).

Sequence analysis of the gp60 locus revealed that all 17 positive isolates were from *C. parvum*. Representative sequences from each identified subtype in this study were deposited in GenBank/EMBl/DDBJ under accession no. KR982672–KR982688. Two *C. parvum* subtype families, IId (11/17) and IIa (6/17), were identified. Within these two *C. parvum* subtype families, two subtypes were each found in each subtype family: IIdA20G1 (7/17) and IIdA26G1 (4/17) in IId and IIaA15G2R1 (5/17) and IIaA16G3R1 (1/17) in IIa.

### Cryptosporidium subtypes and risk factors

The results of the logistic regression analysis of risk factors associated with cryptosporidiosis are shown in Table 1. In the outcome of this model only contact with domestic animals or soil (*P*=0.007, OR = 0.128; CI 95%= 0.029–0.565) and age *(P*=0.001) were identified as the major socio-demographic determinants of *Cryptosporidium* infection.

**Table 1:** Univariate analysis of risk factors associated with frequency of cryptosporidiosis among patients with gastrointestinal disorders from western Iran (n = 1301)

**Variable**	**Positive**	**Negative**	**Total**	**OR**	**CI _95%_**		***P-value***
	**n (%)**	**n (%)**	**n (%)**		**Lower**	**Upper**	
Gender							0.315
Male	11 (1.6)	671 (98.4)	682 (100)	Reference			
Female	6 (0.96)	613 (99)	619 (100)	1.698	0.604	4.773	
Age (Year)							0.001
≤6	7 (1.7)	404 (98.3)	411 (100)	Reference			
7–12	6 (6.7)	83 (93.3)	89 (100)	3.508	1.092	11.261	0.035
> 12	4 (0.5)	797 (99.5)	801 (100)	0.194	0.055	0.680	0.010
Residence							0.057
Rural	13 (1.9)	670 (98.1)	683 (100)	Reference			
Urban	4 (0.6)	614 (99.4)	618 (100)	0.336	0.109	1.035	
Contact with domestic animal & soil							0.007
Yes	14 (2.9)	471 (97.1)	485 (100)	Reference			
No	3 (0.4)	813 (99.6)	816 (100)	0.128	0.029	0.565	
Water supply status							0.057
Untreated (river, well, rain water)	13 (1.9)	670 (98.1)	683 (100)	Reference			
Treated pipe water	4 (0.6)	614 (99.4)	618 (100)	0.336	0.109	1.035	
Seasons							0.082
Spring	3 (0.55)	535 (99.4)	538 (100)	Reference			
Summer	14 (1.8)	749 (98.2)	763 (100)	3.151	0.865	11.479	

All patients (5/5) who were infected with subtype family IIa had contact with domestic animals or soil. Children 7–12 yr were more commonly infected (6.7%) than other age groups (*P*=0.035, OR = 3.508; CI 95%= 1.902–11.261). All subtypes IIa were found in children younger than 10 yr, but IId subtypes were identified in all age groups. Although the majority of *Cryptosporidium*-positive patients were male and lived in rural areas, we did not found any significant association between *Cryptosporidium* infection and residence or gender (*P*>0.05). Moreover, there was no significant association between *Cryptosporidium* infection and season or water supply type (*P*>0.05).

### Clinical features of cryptosporidiosis

Overall, *Cryptosporidium* infection was significantly associated with diarrhea (*P*=0.001, OR=0.092; CI 95%=0.023– 0.362), vomiting & nausea (*P*=0.001, OR=0.013; CI 95%= 0.004–0.044) and abdominal pain (*P*=0.018, OR=0.073; CI 95%=0.008–0.633) in logistic regression analysis. No significant associations were found between cramping (*P*=0.052) or bloating (*P*= 0.746) and *Cryptosporidium* infection (Table 2).

**Table 2:** Clinical features associated with frequency of cryptosporidiosis among patients with gastrointestinal disorders from western Iran (n = 1301)

**Symptoms**	**Samples (n)**	**Positive *Cryptosporidium***	**OR**	**CI _95%_**		***P*-value**
		**n (%)**		**Lower**	**Upper**	
Abdominal pain						0.018
Yes	980	16 (1.6)	Reference			
No	321	1 (0.3)	0.073	0.008	0.633	
Nausea or vomiting						0.001
Yes	58	10 (17.24)	Reference			
No	1243	7 (0.56)	0.013	0.004	0.044	
Crumping						0.052
Yes	523	3 (0.6)	Reference			
No	778	14 (1.8)	4.519	0.990	20.632	
Bloating						0.746
Yes	168	1 (0.6)	Reference			
No	1133	16 (1.4)	1.490	0.133	16.647	
Diarrhea						0.001
Yes	585	13 (2.2)	Reference			
No	716	4 (0.6)	0.092	0.023	0.362	

Among patients infected with the IId sub-type family, 90.1% (10/11) reported abdominal pain, 72.7% (8/11) reported diarrhea and 54.5% (6/11) of patients reported vomiting and nausea. Among those infected with the IIa subtype family, all (6/6) had abdominal pain, 83.3% (5/6) had diarrhea and 50% (3/6) of patients had vomiting and nausea. There was no significant difference between subtype families IIa and IId in the occurrence of clinical symptoms (*P*= 0.75).

## Discussion

The infection rate of *Cryptosporidium* spp. in our study (1.3%) was lower than rates (2.3%–11.5%) reported from previous studies in Iran (4, 11, 15, 16). This difference may be due to differences in geographical locations, study population, and detection methods. The rate of *Cryptosporidium* infection in our study was closer to that reported from children with gastrointestinal illness in Jordan (1.8%) and Philippine (1.9%), and far lower than the rate detected in diarrhea patients in Australia (78%), Ethiopia (20.8%) and Egypt (17%) (17–21).

Findings from the present study revealed that zoonotic transmission of *Cryptosporidium* is common amongst humans in western Iran. Of the 17 isolates that were typed, all were *C. parvum*. This result is similar to recent reports from northern Iran (16). *C. parvum* was the predominant *Cryptosporidium* species in humans and animals (11, 22, 23). However, another study in Iran has identified *C. hominis* (15/21) as the most common species in HIV-positive patients (24). Elsewhere in Middle East countries, *C. parvum* is the predominant *Cryptosporidium* species in humans (19, 25–27).

In this study, sequence analysis of the gp60 locus identified two *C. parvum* subtype families (IIa, IId) and four subtypes (IIaA15G2R1, IIaA16G3R1, IIdA26G1, IIdA20G1). The majority of *Cryptosporidium* infections were caused by IId subtypes (11/17). The IId sub-types have previously been reported commonly in humans in Iran (13, 16) and Kuwait (26), but less frequently in Ethiopia (28), Australia (29) and United Kingdom (30). This subtype family has also reported in sheep and goat in Spain (31) and calves in China, Egypt, and Sweden (32–34). In this study, 72% (8/11) of patients with IId had contact with domestic animals. Two of the subtypes detected in this study (IIdA26G1and IIdA20G1) were previously reported in children in Iran (13). The IIdA20G1 subtype was predominant subtype identified in our study and was previously reported in human in Kuwait and Jordan (19, 26). The subtype IIdA26G1 was previously reported in lambs and goat kids in Spain (31). In addition to IId, subtype family of IIa was also identified in six patients in this study (6/17). IIa is the most prevalent subtype family in animals and human worldwide (32). The IIaA15G2R1 subtype identified in the present study (5/6) is a dominant *C. parvum* subtype in dairy calves around the world (9), supporting the role of zoonotic transmission in cryptosporidiosis in patients in our study. Consistent with this, all IIa patients in the present study had contact with domestic animals. The IIaA16G3R1 subtype was also reported in calves in United States, Ireland and Iran (13, 35–37). Moreover, it was found in humans in Canada and Denmark (35, 38). Our study is first to reporting the IIaA16G3R1 subtype in humans in Iran.

Different *Cryptosporidium* species and sub-types are associated with different clinical symptoms (28, 39). In the present study, *Cryptosporidium* infection was significantly associated with the occurrence of diarrhea, vomiting & nausea and abdominal pain. However, there was no significant difference between the two subtype families in clinical symptoms. In agreement with our results, *C. parvum* infection was associated with diarrhea and vomiting in HIV–infected persons, although in another study (40) they reported that *C. parvum* infection was associated only with diarrhea in children (39). Similar results were obtained from Ethiopia, where *C. parvum* especially IIa sub-type, family was associated only with the occurrence of diarrhea (28). The role of parasite genetics in clinical manifestations of cryptosporidiosis is still not clear and further studies are needed to elucidate fully the characteristics of this association.

Results of the risk factor analysis support the role of zoonotic transmission in *Cryptosporidium* epidemiology in patients in western Iran. Among infected patients, 82.3% (14/17) reported contacted with domestic animals. Another significant risk factor in our study was age. We found that all patients with IIa sub-type family infection were younger than ten years, while those infected patients with IId subtype family were in different age groups (8–45 yr). These results are consistent with previous studies in Iran and elsewhere (13, 16, 17, 28, 30, 41).

## Conclusion

Cryptosporidiosis may be an important cause of gastrointestinal illnesses, especially among children. Moreover, *C. parvum* is the main species in Nahavan County, west of Iran, suggesting that zoonotic transmission is main route in the acquisition of cryptosporidiosis infection in this region. Therefore, improved hygiene and avoidance of contact with animals and contaminated soil should be advocated to reduce the occurrence of *Cryptosporidium* infections, especially in children. Further investigations are needed to elucidate fully possible difference in clinical presentations among *Cryptosporidium* species and major subtypes.
